# Definition of CRISPR Cas12a *Trans*-Cleavage Units to Facilitate CRISPR Diagnostics

**DOI:** 10.3389/fmicb.2021.766464

**Published:** 2021-11-29

**Authors:** Hailong Lv, Jian Wang, Jian Zhang, Yijian Chen, Lei Yin, Dian Jin, Dayong Gu, Huailong Zhao, Yong Xu, Jin Wang

**Affiliations:** ^1^Department of Clinical Laboratory, Shenzhen Institute of Translational Medicine, The First Affiliated Hospital of Shenzhen University, Shenzhen Second People’s Hospital, Shenzhen, China; ^2^Department of Biomedical Engineering, Guangdong Key Laboratory for Biomedical Measurements and Ultrasound Imaging, School of Medicine, Shenzhen University, Shenzhen, China; ^3^Institute of Antibiotics, Huashan Hospital, Fudan University, Shanghai, China; ^4^Key Laboratory of Clinical Pharmacology of Antibiotics, National Health Commission, Shanghai, China; ^5^Tolo Biotechnology Company Limited, Shanghai, China; ^6^TEDA Campus of Tianjin University of Science and Technology, Binhai New Area Development Zone, Tianjin, China; ^7^Jinan Center for Disease Control and Prevention, Jinan, China; ^8^Guangdong Provincial Key Laboratory of Systems Biology and Synthetic Biology for Urogenital Tumors, The First Affiliated Hospital of Shenzhen University, Shenzhen Second People’s Hospital (Shenzhen Institute of Translational Medicine), Shenzhen, China

**Keywords:** CRISPR diagnostics, *trans*-cleavage, enzymatic units, Cas12, *trans*U

## Abstract

The CRISPR diagnostic (CRISPR-Dx) technology that employs the *trans*-cleavage activities has shown great potential in diagnostic sensitivity, specificity, convenience, and portability, and has been recognized as the next-generation diagnostic methods. However, due to the lack of standardized definition of Cas *trans*-cleavage enzymatic units, it is difficult to standardize the present CRISPR-Dx systems, which have undoubtedly impeded the development of the CRISPR-Dx industry. To solve the problem, we here first systematically optimized the reaction systems for Cas12a, and then defined its *trans*-cleavage units (*trans*U), which we believe will be of great importance and interest to researchers in both molecular diagnostic industry and basic research. Moreover, a simple protocol was provided to facilitate a step-by-step measurement of the Cas12a *trans*U, which can also act as a reference for the definition of the *trans*U for other Cas proteins.

## Introduction

The recently characterized *trans*-cleavage activities of the Clustered Regularly Interspaced Short Palindromic Repeats (CRISPR) Cas enzymes such as Cas12 and Cas13 has undoubtedly sparked the interests in developing CRISPR diagnostic (CRISPR-Dx) systems (Gootenberg et al., 2017, 2018; Chen et al., 2018; Li et al., 2018b; Li Y. et al., 2019). The *trans*-cleavage activities of Cas12a were first discovered during the characterization of its cleavage behaviors against target single-stranded DNA (ssDNA) (Chen et al., 2018; Li et al., 2018a; Li Y. et al., 2019). Under the guidance of CRISPR RNA (crRNA), Cas12a recognizes target DNA, forms a ternary complex of Cas12a, crRNA, and target DNA, and then *trans*-cleaves non-specific ssDNA in the system, which activity is designated as the *trans*-cleavage activity (Chen et al., 2018; Li et al., 2018a). Similar ssDNA *trans*-cleavage activities have been found in other Cas12 subtypes such as the thermophilic Cas12b and the Cas12f (previously known as Cas14) that specifically recognizes ssDNA targets (Leung et al., 2021). With the employment of these Cas12 *trans*-cleavage activities, dozens of CRISPR-Dx systems have been successfully created, among which HOLMES (one-hour low-cost multipurpose highly efficient system), HOLMESv2 and DETECTR (DNA Endonuclease Targeted CRISPR *Trans* Reporter) are the representatives (Chen et al., 2018; Li et al., 2018b; Li L. et al., 2019).

HOLMES first uses the polymerase chain reaction (PCR) technology to amplify the target nucleic acids and then detects the amplicons with the Cas12a *trans*-cleavage activities, illuminating the fluorescent signals. HOLMES detects target nucleic acid sequences with the attomolar sensitivity and distinguishes single-base mismatches (Li et al., 2018b). However, the PCR amplification and Cas12a *trans*-cleavage processes are separated in HOLMES and the transfer of amplicons may result in aerosol contamination. To solve the problem, the physically separated amplification and Cas12a *trans*-cleavage steps may either be designed in a closed microfluidic system or operated in a standard PCR laboratory that contains separate rooms for distinct purposes; however, it may cause inconvenience in use of the HOLMES technology. Alternatively, HOLMESv2 integrates the thermophilic Cas12b with the Loop-mediated isothermal amplification (LAMP) and supports one-pot diagnosis of target nucleic acids (Li L. et al., 2019). Unlike Cas12, Cas13 targets RNA and is triggered to *trans*-cleave collateral single-stranded RNA reporter sequences in the system, with which diagnostic systems such as SHERLOCK (Specific High Sensitivity Enzymatic Reporter UnLOCKing) (Gootenberg et al., 2017) were developed. Then, with the employment of Cas12 and Cas13, SHERLOCKv2 was created to simultaneously detect multiple target nucleic acids in one diagnostic system (Gootenberg et al., 2018). Recently, with the use of either tandem crRNAs or remarkably reduced reaction volumes for target nucleic acid detection, diagnostic sensitivities can be greatly improved, and several amplification-free systems have been successfully developed (Fozouni et al., 2021; Liu et al., 2021; Shinoda et al., 2021; Tian et al., 2021; Yue et al., 2021).

In comparison with traditional molecular diagnostic methods such as polymerase chain reaction (PCR), CRISPR-Dx methods have shown great advantages in sensitivity, specificity, simplicity and portability and have been well recognized as the next-generation diagnostic methods (Chertow, 2018). Therefore, soon after the outbreak of the COVID-19 pandemic, a large number of diagnostic methods were successfully developed, most of which employed the immunochromatography and the real-time PCR technologies and have played an important role in the epidemic prevention and control. Meanwhile, dozens of CRISPR-Dx methods were also developed for COVID-19 diagnosis, including the one-pot systems that combine either recombinase polymerase amplification (RPA) with Cas12a (Aman et al., 2020; Ding et al., 2020) or LAMP with Cas12b (Joung et al., 2020), all showing advantages in diagnostic sensitivity, specificity, and convenience (Han et al., 2021). Noticeably, within 3 years since the first report of the CRISPR-Dx technology in 2017 (Gootenberg et al., 2017), the United States Food and Drug Administration (FDA) granted the Emergency Use Authorization (EUA) to Sherlock biosciences for the CRISPR SARS-CoV-2 rapid diagnostic kit (Martin et al., 2021), which once again confirmed the great potential of CRISPR technologies in pathogen diagnosis.

However, due to the lack of unified *trans*-cleavage units (*trans*U), the Cas enzymes used in present systems are usually quantitated in concentration instead of enzymatic units. As the Cas specific *trans*-cleavage activities may vary from different commercial providers, distinct diagnostic performances can be obtained for a defined CRISPR-Dx system if the Cas enzyme is definitized in concentration, which will undoubtedly limit large-scale industrial applications of CRISPR-Dx technologies. Besides, it is well known that enzymatic cleavage activities can be affected by many reaction factors, including ions, salt concentration, *pH* values and temperature. Therefore, we here systematically studied the factors affecting Cas12a *trans*-cleavage activities, optimized the reaction systems and finally defined its *trans*U. Moreover, a protocol was provided to simplify the *trans*U definition procedures.

## Results and Discussion

### Optimization of the Cas12a *Trans*-Cleavage Reporters

To precisely calculate the Cas12a *trans*-cleavage activity, the ssDNA reporter used was dual labeled with fluorophore and quencher (i.e., FQ-reporter), and the reaction was monitored by a fluorescence reader. To simplify the calculation, the *trans*-cleavage activity was calculated on the basis of the initial fluorescence growth rate *v*_*g*_ (*v*_*g*_ = ΔF/Δt), following the recently published Michaelis-Menten model (Ramachandran and Santiago, 2021).

**FIGURE 1:**
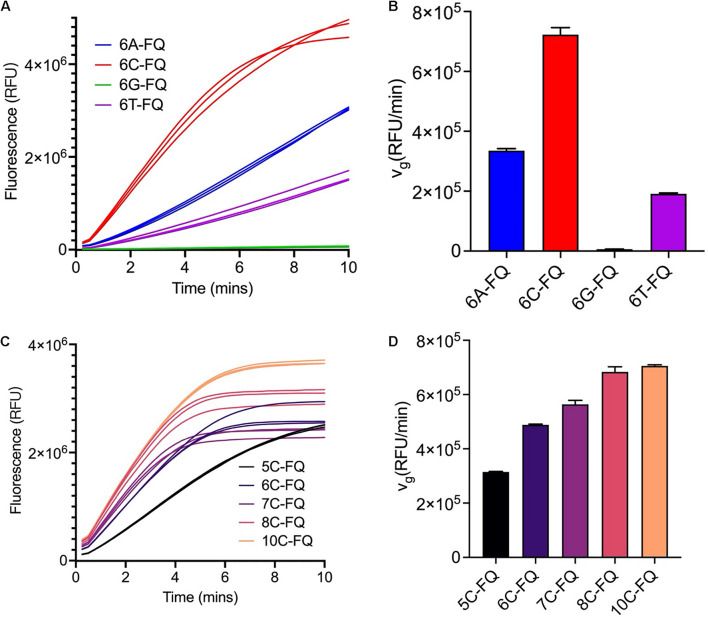
Optimization of the ssDNA reporter sequences. **(A)** Fluorescence curves of the Cas12a *trans*-cleavage reactions using different ssDNA reporter sequences. The reaction systems were comprised of 250 nM crRNA, 250 nM Cas12a, 3 nM target dsDNA, 1000 nM reporter and 1× NEBuffer 3.1. **(B)** Initial fluorescence growth rate of the reporters used in panel **(A)**. **(C)** Fluorescence curves of the Cas12a *trans*-cleavage reactions using poly-C ssDNA reporters of different lengths. The reaction systems were comprised of 250 nM crRNA, 250 nM Cas12a, 3 nM target dsDNA, 800 nM reporter, and 1× NEBuffer 3.1. **(D)** Initial fluorescence growth rate of the reporters used in panel **(C)**.

Non-paired ssDNA FQ-reporters are the substrates for Cas12a *trans*-cleavage reactions, and the sequences may affect the cleavage efficiencies (Li et al., 2018a). We first tested four homopolymers of 6 nucleotides in length, including poly-adenine (A), poly-cytosine (C), poly-guanine (G), and poly-thymine (T), respectively, and found Cas12a *trans*-cleaved the poly-C reporter with the highest efficiency but failed to cleave the poly-G reporter (Figures 1A,B), which was consistent with the previous findings (Yue et al., 2021). Along with the elongation of the ssDNA reporter sequence, the *v*_*g*_ gradually increased and reached the peak when the sequence was longer than 8 nucleotides (Figures 1C,D). Considering the fact that longer ssDNA FQ-reporter has higher background fluorescence and is more expensive to synthesize, the 8C FQ-reporter was chosen for the following assays.

### Optimization of the Cas12a *Trans*-Cleavage Reaction Buffer

**FIGURE 2:**
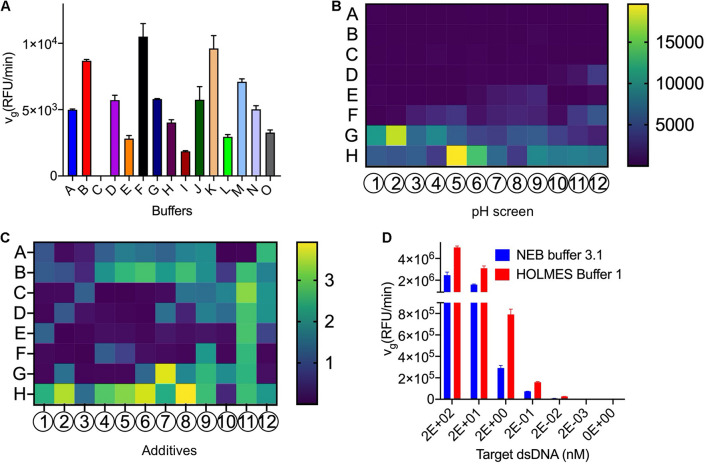
Optimization of the Cas12a *trans*-cleavage reaction buffer. **(A)** Screening of the commercially available buffers. Detailed compositions of the tested buffers were listed in Supplementary Table 1. **(B)** Screening of the *pH* values with the *pH* slice plate (Cat. No. #HR2-070) from Hampton Research. The A–H and 1–12 indicated the positions in the 96-well plate and detailed components in each well were shown in Supplementary Sheet 1. Specifically, Cas12a in conditions of H5 (*pH* 8.6 in 1 M glycine buffer) and G2 (*pH* 8.5 in 1 M Tris–HCl buffer) showed obviously higher *trans*-cleavage activities and the Tris buffer with *pH* 8.5 was finally chosen for further analysis. The *trans*-cleavage activities (RFU/min) were reflected by the colored histogram on the right side. **(C)** Screening of additives with the plate (Cat. No. #HR2-072) from Hampton Research. The A–H and 1–12 indicated the positions in the 96-well plate and detailed components in each well were shown in Supplementary Sheet 2. In comparison with the water control in A1, the Cas12a showed obviously higher *trans*-cleavage activities with several additives such as the G7 (glycerol), H5 (PEG 3,350), H6 (PEG 8,000), and H8 (PEG 20,000), and the most efficient H8 was employed for further analysis. The fold changes, relative to the activities in A1, were indicated by the colored histogram on the right side. **(D)** Comparison of the *v*_*g*_ of Cas12a in HOLMES Buffer 1 and NEB buffer 3.1.

To optimize the Cas12a reaction buffer, we first compared several commercially available reaction buffers and found that Cas12a in buffer F had the highest *trans*-cleavage efficiencies (Figure 2A and Supplementary Table 1). As the *pH* values may have influences on the Cas12a *trans*-cleavage activities, a group of commercially available standard buffers with *pH* values ranging from 3.5 to 9.6 in increments of 0.1 were further analyzed, through individually substituting the (hydroxymethyl)aminomethane (abbreviated as Tris) salt in buffer F. Among the tested conditions, Cas12a generally preferred buffers with high *pH* values and exhibited obviously higher *trans*-cleavage activities in the 1 M glycine buffer (*pH* 8.6) and the 1 M Tris–HCl buffer (*pH* 8.5) (Figure 2B). Considering the buffering capacity in maintaining a stable *pH* value of the reaction buffers, especially when complicated samples are detected, the Tris–HCl buffer (*pH* 8.5) was finally chosen. Meanwhile, the loss of Cas12a *trans*-cleavage activities in buffer C could be caused by either the relatively low *pH* value or the low concentration of Mg^2+^, which has been demonstrated to be important for the maintenance of Cas12a *cis*-cleavage activities in a previous study (Fonfara et al., 2016; Supplementary Table 1).

Meanwhile, dozens of additives were individually added into the reaction buffer F to test their efficacy on the Cas12a *trans*-cleavage activities, and several components such as polyethylene glycol (PEG) and glycerol obviously enhanced the cleavage signals and were chosen for further analysis (Figure 2C). The chosen additives of different combinations and concentrations were compared and the condition of 0.4% PEG-20000 showed the most remarkable enhancement and was finally selected (Supplementary Figure 1). In addition, we found that non-ionic detergents such as Triton X-100 could also promoted the Cas12a *trans* activities.

After systematical analysis of the above selected components, an optimized reaction buffer namely HOLMES Buffer 1 showed better performance than buffer F in triggering the Cas12a *trans*-cleavage and was chosen for subsequent analysis (Supplementary Figure 2 and section “5.3.1” in Supplementary Protocol). Moreover, through comparison with the control buffer of NEB buffer 3.1, HOLMES Buffer 1 remarkably enhanced the Cas12a *trans*-cleavage activities as well as its detection sensitivities (Figure 2D).

Besides the reaction buffer, the ratio among Cas12a, crRNA, and target dsDNA could also be an important factor affecting the Cas12a *trans*-cleavage efficiencies, and different combinations were therefore tested in HOLMES Buffer 1. In theory, excessive amounts of crRNA and target dsDNA could promote the formation of the Cas12a ternary complex as well as triggering of the Cas12a *trans*-cleavage; however, we found that the Cas12a cleavage activity was slightly inhibited by high concentrations of crRNA and dsDNA with an unknown reason (Supplementary Figure 3). Therefore, a ratio of 1: 2: 2 was finally chosen to obtain the best kinetic parameters for Cas12a *trans*-cleavage in HOLMES Buffer 1.

### Measurement of the Cas12a *Trans*-Cleavage Enzymatic Constants

**FIGURE 3:**
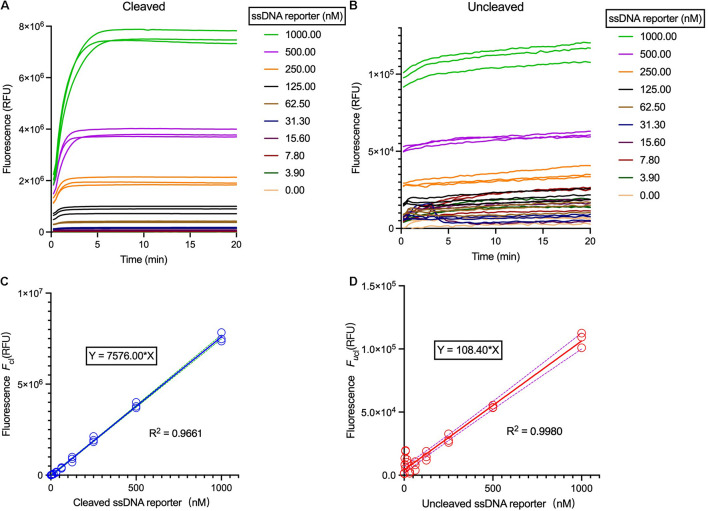
Correlation of the relative fluorescent units (RFUs) and the FQ-reporter concentration. **(A)** Fluorescence curves of the Cas12a *trans*-cleavage reactions using different ssDNA reporter concentration. **(B)** Fluorescence curves of the Cas12a *trans*-cleavage reactions using different ssDNA reporter concentration without target. The reaction systems were comprised of 40 nM crRNA, 20 nM Cas12a, 40 nM target dsDNA, and 1× HOLMES Buffer 1. **(C)** Background-subtracted fluorescence *F*_*cl*_ versus concentration of cleaved reporters. **(D)** Background-subtracted fluorescence *F*_*ucl*_ versus concentration of un-cleaved reporters. Solid lines are linear regression fits to experimental data (in symbols). Three replicates were measured for each concentration. Both cleaved and un-cleaved reporters were detected by the Applied Biosystems QuantStudio 3 machine.

At the presence of the target DNA, the Cas12a *trans*-cleaves the ssDNA FQ-reporter to illuminate fluorescent signals, which can be employed to measure the Cas12a enzymatic constants. However, as the fluorescence intensities may vary from different readers, standard curves should be first created to correlate the relative fluorescent units (RFUs) and the concentrations of the FQ-reporter, including both un-cleaved (i.e., the substrates) and the cleaved (i.e., the products) reporters (Figures 3A,B and Supplementary Figures 4a,b). Briefly, the ssDNA FQ-reporter should be serially diluted and both cleaved and un-cleaved fluorescence signals are continually measured till the fluorescence reaches the saturation point, and the highest values of each gradient are then used for calibration of the standard curves for both cleaved and un-cleaved reporters (Figures 3C,D and Supplementary Figures 4c,d).

**FIGURE 4:**
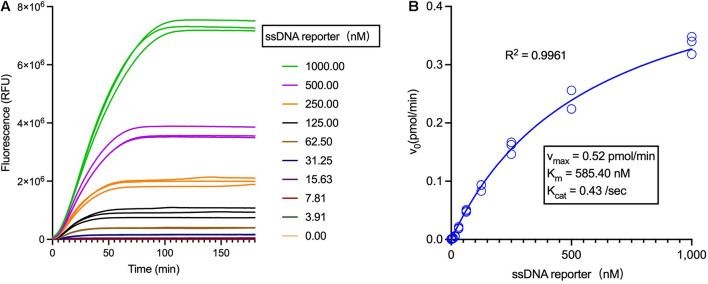
Analysis of the LbCas12a enzymatic kinetics. **(A)** Fluorescent curves of the Cas12a *trans*-cleavage reactions using different concentrations of the ssDNA reporter. **(B)** Analysis of the Michaelis-Menten constants of LbCas12a (Tolo Biotech.), employing 1 nM Cas12a and varied concentrations of ssDNA reporters.

Then, with the employment of a limited concentration of Cas12a and crRNA and varied concentrations of ssDNA reporters, *trans*-cleavage enzymatic kinetics for Cas12a were determined, including the values of *v*_max_, *K*_m_, and *K*_cat_ (Figure 4 and Supplementary Table 2). Noticeably, the constants of *K*_m_, *K*_cat_ as well as the *K*_cat_/*K*_m_ were miscalculated in a previous study (Chen et al., 2018), which were much higher than the data in this study and another work (Ramachandran and Santiago, 2021) and have been recently corrected (Supplementary Table 3).

### Definition of the Cas12a *Trans*-Cleavage Units

In the above-mentioned optimized reaction condition, the Cas12a *trans*-unit (*trans*U) was defined as the amount of Cas12a that *trans*-cleaves 1 pmol 8C FQ-reporter in 1 min at 37°C in a total reaction volume of 20 μL in HOLMES Buffer 1. To simplify the *trans*U measurement, a step-by-step protocol was provided in the Supplementary Protocol, which can be run on different fluorescence readers. Noticeably, as the Cas12a *trans*-cleavage efficiencies can be remarkably affected by the crRNA and target dsDNA sequences as well as their concentrations, the *trans*U should be determined using the same sequences and concentrations as provided in this study to unify the measurement (*ref to* sections “5 and 6.2” in Supplementary Protocol).

**FIGURE 5:**
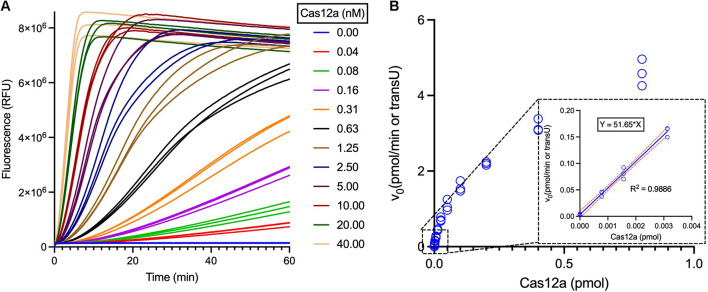
Measurement of the LbCas12a specific *trans*-cleavage activities. **(A)** Fluorescent curves of the Cas12a *trans*-cleavage reactions with different concentrations of LbCas12a. LbCas12a was twofold serially diluted for the *trans*U measurement, using 1 μM ssDNA reporter as the substrate and following the Supplementary Protocol. **(B)** Curve plotting of calculated initial reaction rate *versus* the LbCas12a concentrations. The linear region of the reactions was boxed and enlarged, and the data were further employed for *trans*U calculation.

Briefly, a standard curve correlating the RFUs and the reporter concentrations should be drawn first, and then the *trans*-cleavage reaction is performed with serially diluted Cas12a and excess ssDNA reporters. The obtained data in the linear region are then used for calculation of the Cas12a *trans*U. Following this protocol, a commercially available LbCas12a was then analyzed, and the *trans*-cleavage activities were determined to be 51.65 *trans*Us/pmol (i.e., 19.36 fmol per *trans*U) (Figure 5).

Taken together, we here systematically optimized the Cas12a *trans*-cleavage reaction conditions, measured the Cas12a enzymatic constants and defined its *trans*U, which will undoubtedly facilitate the development and application of robust Cas12a-based CRISPR-Dx systems in the future. Moreover, following a similar approach, the *trans*U of other Cas effectors such as Cas12b, Cas12f, and Cas13 can be defined and their measurement protocols can be easily developed. On the other hand, although Cas12a exhibits much better performance in HOLMES Buffer 1 (e.g., with a higher *K*_cat_ value), one should be aware that there is still much work to do to further improve the Cas12a *trans*-cleavage activities, which may include the optimization of not only the reaction buffers but also the crRNA sequences in the future.

## Materials and Methods

### Cas Proteins, Oligos, and Reagents

LbCas12a (LbCpf1) (Cat. No. #32108-03) was ordered from Tolo Biotech. (Shanghai, China). crRNAs were chemically synthesized by Biolino Acid Technology (Tianjin, China). FQ-labeled ssDNA reporters were ordered from Sangon Biotech. (Shanghai, China). The additive screening plates (Cat. No. #HR2-072) and the *pH* slice plate (Cat. No. #HR2-070) were ordered from Hampton Research (Aliso Viejo, CA).

### Preparation of Target dsDNA

For the short dsDNA target, two complementary oligonucleotides were synthesized by commercial companies and then annealed to form dsDNA. Specifically, a pair of oligonucleotides (4 μM each) were mixed in 1× PCR buffer and then annealed in the following procedure with a thermal cycler: 95°C for 5 min and then reduced from 95 to 20°C at a rate of 1°C per minute. The annealed dsDNA target was diluted to 1 μM and stored at −20°C before use.

### Screening of the Cas12a *Trans*-Cleavage Additives

The Cas12a *trans*-cleavage reaction was performed in a 20-μL volume, following the conditions as described before (Li et al., 2018b). In detail, 50 nM crRNA, 5 nM Cas12a nuclease, 4 nM dsDNA target, and 200 nM 8C FQ-reporter were pre-mixed in 1× buffer F and then 16 μL of the mixture was aliquoted into the 96-well PCR plate, which should be operated on ice. Then, the reaction was initiated by adding 4-μL additives into each well and the fluorescence signals were measured by the qPCR machine (Applied Biosystems QuantStudio 3). The program was set at 37°C and signals (λex: 488 nm; λem: 535 nm) were collected every 15 s. The initial fluorescence growth rate (*v*_*g*_) was calculated according to the maximum slope of the fluorescence curve using the ICEKAT software^1^.

### Michaelis-Menten Analysis

The Michaelis-Menten was analyzed according to following equation: *v*_0_ = *v*_max_ [S]/(*K*_m_ + [S]), where [S] is the substrate concentration, *v*_max_ is the maximum reaction rate, and *K*_m_ is the Michaelis constant. The turnover number (*K*_cat_) was determined by the equation: *K*_cat_ = *v*_max_/[Et], where Et was the effective complex. The concentration of the activated Cas12a enzymatic complex was always kept at 1 nM in 1× reaction buffer. The Cas12a *trans*-cleavage reaction was initiated by the addition of the ssDNA FQ-reporter at the final concentrations of 0 nM, 3.9 nM, 7.8 nM, 15.6 nM, 31.2 nM, 62.5 nM, 125 nM, 250 nM, 500 nM, and 1 μM, respectively. Reactions were carried out in three replicates at 37°C and fluorescence readouts were taken every 15 s. Background-subtracted fluorescence signals were obtained by subtracting the signal from a negative control. The *v*_0_ data were converted to nM/s from Relative Fluorescent Units using the calibrated standard curve (*ref to* section “6.1” in Supplementary Protocol), and the Michaelis-Menten constants were determined by plotting *v*_0_ against the substrate concentration and then fitting into the Michaelis-Menten curve (Graphpad Software).

## Data Availability Statement

The original contributions presented in the study are included in the article/Supplementary Material, further inquiries can be directed to the corresponding authors.

## Author Contributions

HL, JianW, and JZ performed most of the experiments. YC, LY, and DJ provided assistances in defining the Cas12a *trans-*cleavage units. HL and JinW drafted the manuscript. DG, HZ, YX, and JinW analyzed the data, revised the manuscript, and supervised the study. All authors contributed to the article and approved the submitted version.

## Conflict of Interest

LY is employed by Tolo Biotechnology Company Limited, Shanghai, China. The remaining authors declare that the research was conducted in the absence of any commercial or financial relationships that could be construed as a potential conflict of interest.

## Publisher’s Note

All claims expressed in this article are solely those of the authors and do not necessarily represent those of their affiliated organizations, or those of the publisher, the editors and the reviewers. Any product that may be evaluated in this article, or claim that may be made by its manufacturer, is not guaranteed or endorsed by the publisher.

## Footnotes

1
https://icekat.herokuapp.com/icekat
