# Supplementary material for: Definition of CRISPR Cas12a Trans-Cleavage Units to Facilitate CRISPR Diagnostics
Source: Front Microbiol. 2021 Nov 29;12:766464. doi: 10.3389/fmicb.2021.766464 (PMC8667580; doi:10.3389/fmicb.2021.766464)
Supplement: Supplementary file 1 [file Data_Sheet_1.DOCX]

**Definition of the Cas12a *trans*-cleavage unit to facilitate CRISPR diagnostics**

**Supplementary Figures**


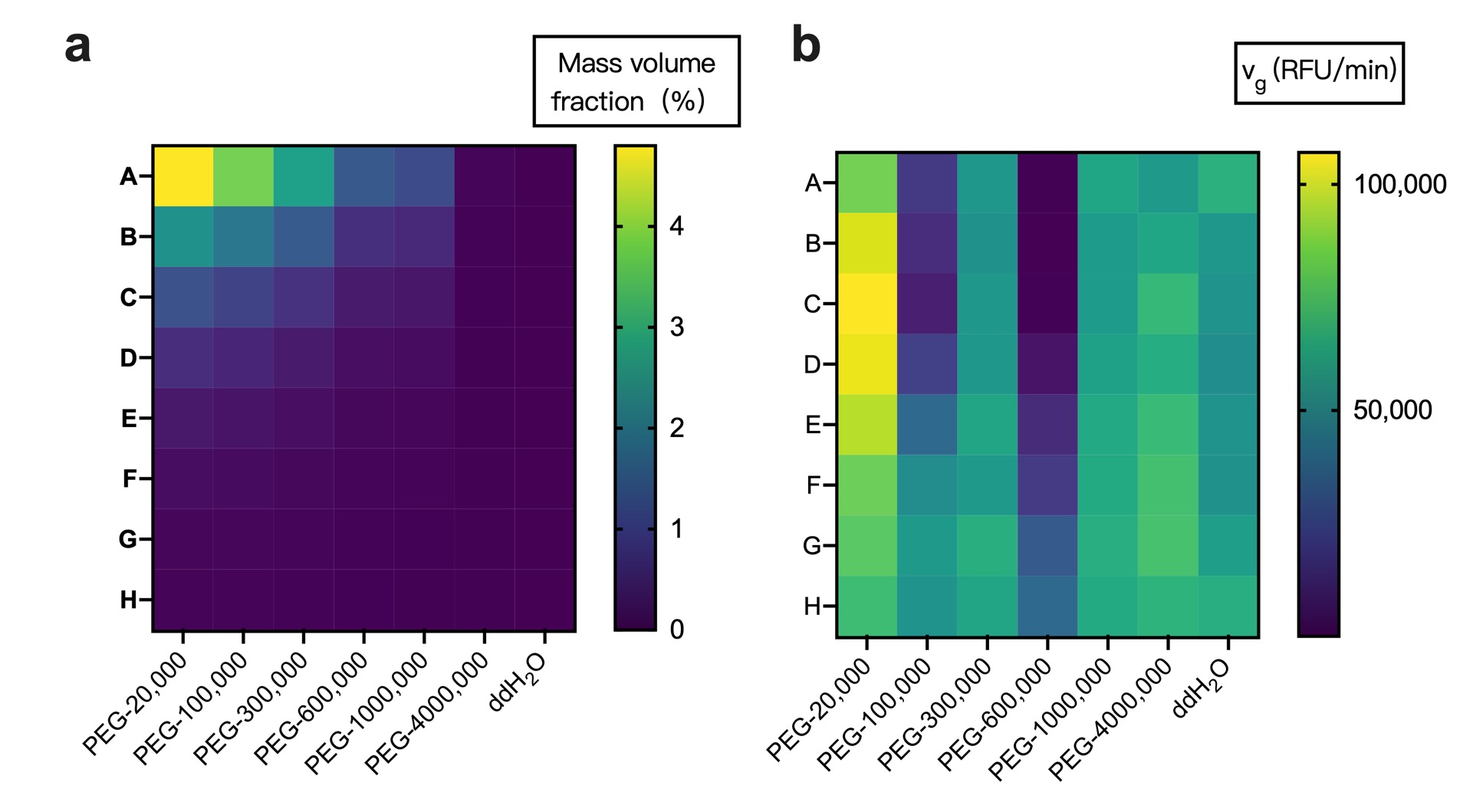


**Figure S1. Screening of PEGs for Cas12a *trans*-cleavage reaction systems.** (a) PEGs of different sizes and different concentrations were tested. (b) The initial fluorescence growth rate (v_g_) of the reactions in panel a.


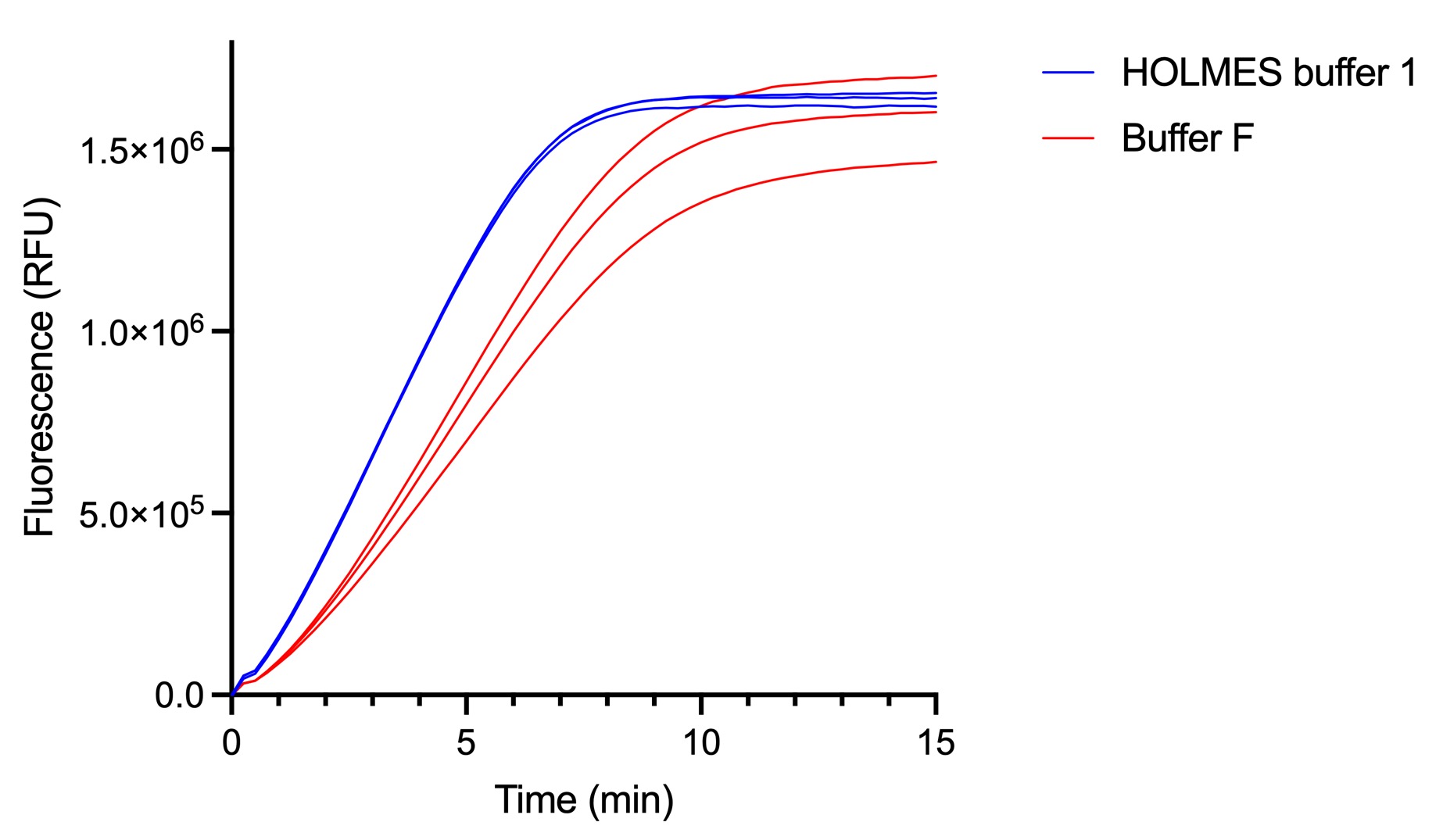


**Figure S2. Comparison of the Cas12a *trans*-cleavage activities in different reaction buffers.** The HOLMES Buffer 1 was obtained on the basis of buffer F and showed better performance in triggering the Cas12a *trans*-cleavage reactions.


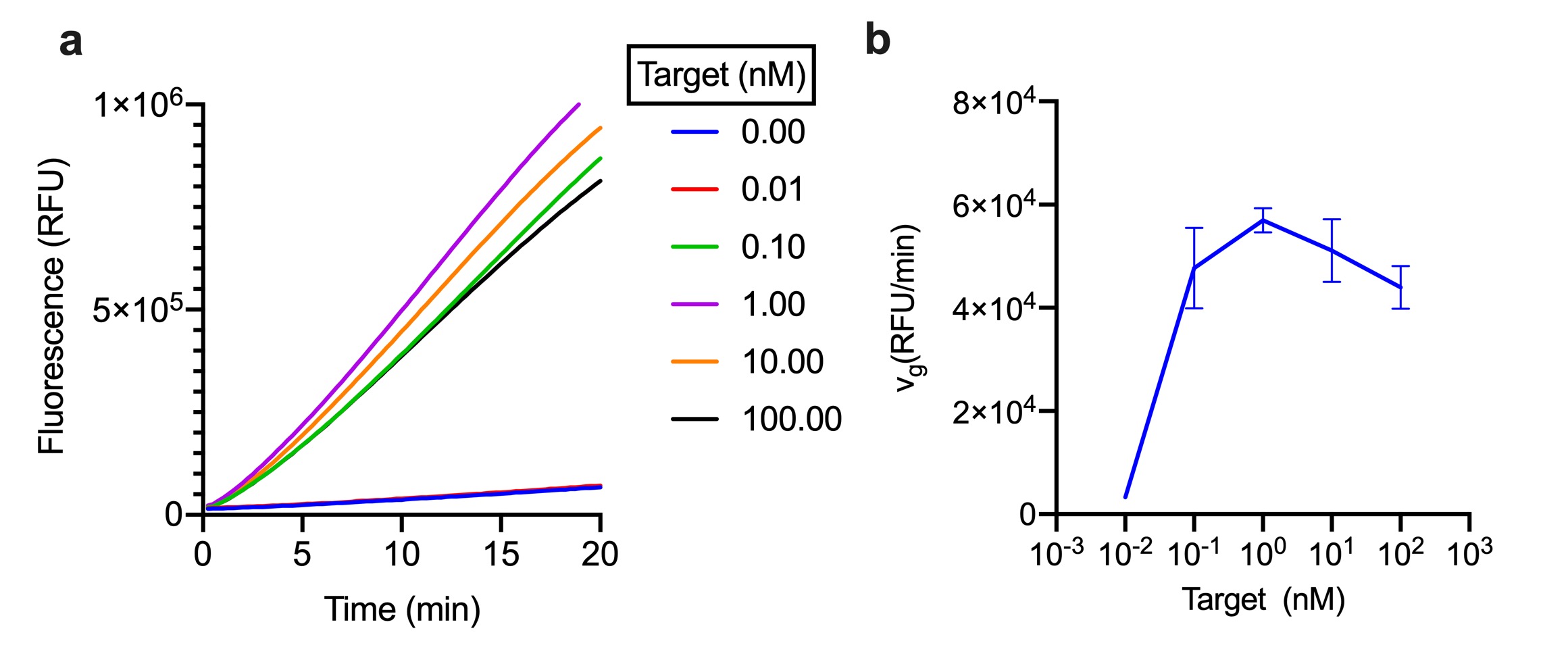


**Figure S3. The influence of the concentrations of the target dsDNA on Cas12a *trans*-cleavage activities.** (a) The fluorescence curves of the Cas12a *trans*-cleavage reactions, employing different concentrations of target dsDNA. (b) The v_g_ values of the reactions using target dsDNA with the corresponding concentrations as shown in panel a.


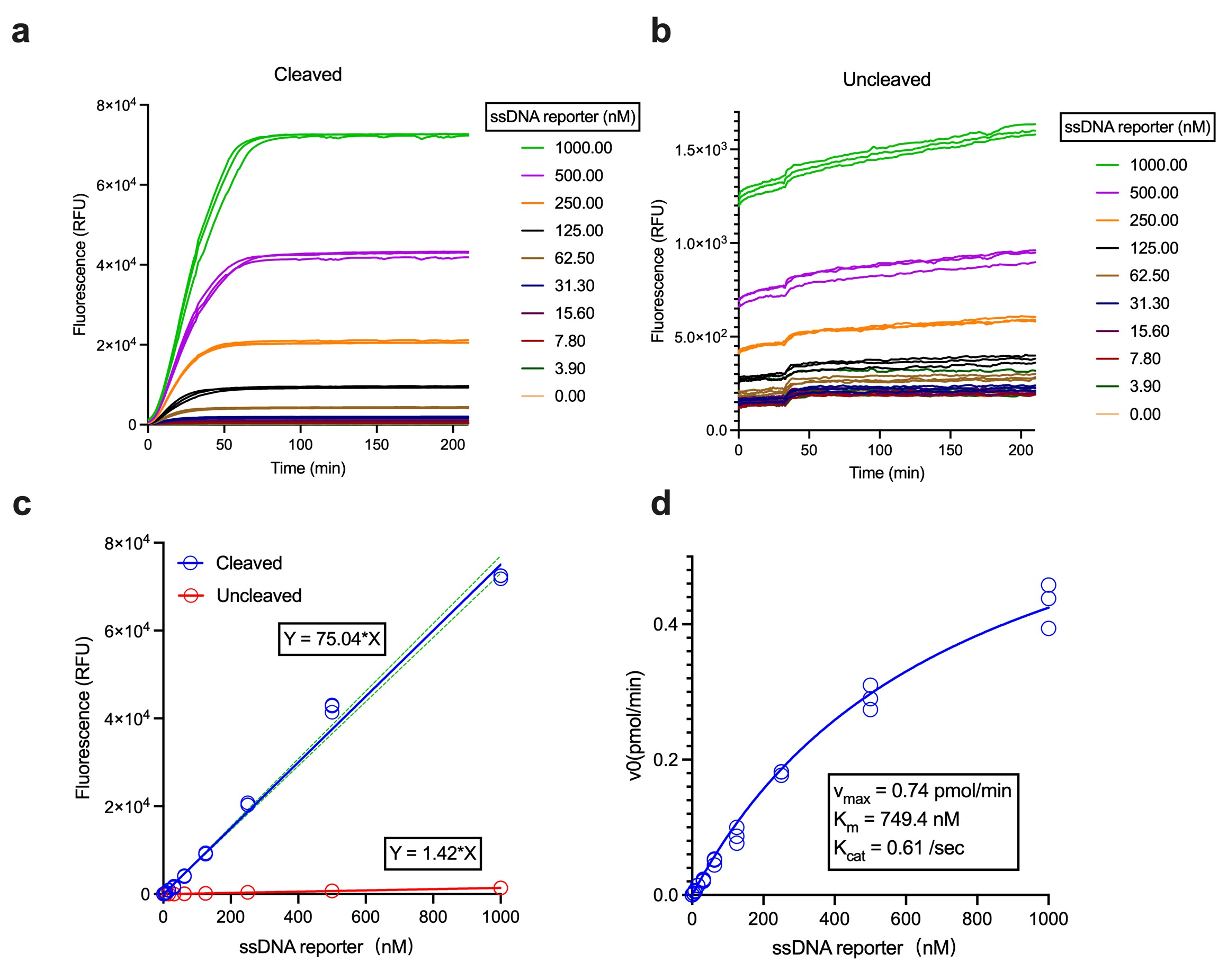


**Figure S4. Analysis of the Cas12a *trans*-cleavage kinetics using a qPCR machine from a different provider.** The *trans*-cleavage reaction systems were the same as shown in the **Supplementary Protocol** and all reactions were performed in a total volume of 20 μL with the fluorescence signals recorded by the LineGene 9600 qPCR system (Hangzhou Bioer, China). (a) Fluorescence curves of Cas12a *trans*-reactions using different concentrations of the ssDNA FQ-reporter, which was cleaved by 1 nM Cas12a in the HOLMES Buffer 1. (b) Fluorescence curves of the negative control experiments (NTC) corresponding to reaction systems shown in panel a. In NTC, the Cas12a enzyme was substituted with nuclease-free water, representing the un-cleaved signals. (c) Calibrated curve with the background-subtracted fluorescence signals *versus* the concentrations of cleaved and un-cleaved reporters. (d) Measurement of the Michaelis-Menten *trans*-cleavage kinetics for LbCas12a (Tolobio). Reactions were performed with different concentrations of the FQ-reporter, using 1 nM activated ternary complex of Cas12-crRNA-dsDNA. Three replicates were performed for each reaction condition and a Michaelis−Menten equation was obtained (*ref to* the solid line).

**Supplementary Tables**

**Table S1. Compositions of the tested buffers.**

| **No.** | **Names/Cat. No.s** | **Provider** | **Components** |
| --- | --- | --- | --- |
| A | TOLO Buffer 3 (#32104-03) | Tolo Biotechnology | N/A |
| B | DNase I Reaction Buffer (#B0303S) | New England Biolabs | 10 mM Tris-HCl; 2.5 mM MgCl_2_; 0.5 mM CaCl_2_; pH 7.6@25°C |
| C | Antarctic Phosphatase Reaction Buffer (#B0289) | New England Biolabs | 50 mM Bis-Tris-Propane-HCl; 1 mM MgCl_2_; 0.1 mM ZnCl_2_; pH 6@25°C |
| D | T4 DNA Ligase Reaction Buffer (#B0202) | New England Biolabs | 50 mM Tris-HCl; 10 mM MgCl_2_; 1 mM ATP; 10 mM DTT; pH 7.5@25°C |
| E | StickTogether™ DNA Ligase Buffer (#B0535) | New England Biolabs | 66 mM Tris-HCl; 10 mM MgCl_2_; 1 mM ATP; 1 mM DTT; 7.5% Polyethylene glycol (PEG 6000); pH 7.6@25°C |
| F | RNAPol Reaction Buffer (#B9012) | New England Biolabs | 40 mM Tris-HCl; 6 mM MgCl_2_; 1 mM DTT; 2 mM spermidine; pH 7.9@25°C |
| G | T4 Polynucleotide Kinase Reaction Buffer (#B0201) | New England Biolabs | 70 mM Tris-HCl; 10 mM MgCl_2_; 5 mM DTT; pH 7.6@25°C |
| H | ThermoPol®Reaction Buffer Pack (#B9004) | New England Biolabs | 20 mM Tris-HCl; 10 mM (NH4)_2_SO_4_; 10 mM KCl; 2 mM MgSO_4_; 0.1% Triton® X-100; pH 8.8@25°C |
| I | Exonuclease I Reaction Buffer (#B0293) | New England Biolabs | 67 mM Glycine-KOH; 6.7 mM MgCl_2_; 10 mM β-ME; pH 9.5@25°C |
| J | EasyTaq Buffer (#AP111) | Transgen Biotechnology | 10 mM (NH4)_2_SO_4_; 20 mM KCl; 2 mM MgSO_4_; 20 mM Tris-HCl; pH 8.3@25°C |
| K | CutSmart® Buffer (#B7204) | New England Biolabs | 50 mM KAc; 20 mM Tris-acetate; 10 mM MgAc_2_; 100 µg/ml BSA; pH 7.9@25°C |
| L | NEBuffer™ 3.1 (#B7203) | New England Biolabs | 100 mM NaCl; 50 mM Tris-HCl; 10 mM MgCl_2_; 100 µg/ml BSA; pH 7.9@25°C |
| M | NEBuffer™ 2.1 (#B7202) | New England Biolabs | 50 mM NaCl; 10 mM Tris-HCl; 10 mM MgCl_2_; 100 µg/ml BSA; pH 7.9@25°C |
| N | NEBuffer™ 2 (#B7002S) | New England Biolabs | 50 mM NaCl; 10 mM Tris-HCl; 10 mM MgCl_2_; 1 mM DTT; pH 7.9@25°C |
| O | NEBuffer™ 1.1 (#B7201) | New England Biolabs | 10 mM Bis-Tris-Propane-HCl; 10 mM MgCl_2_; 100 µg/ml BSA; pH 7@25°C |

**Table S2. The *trans*-cleavage kinetic parameters of LbCas12a from Tolo Biotech.**

| **Parameters** | **Value** | **Unit** |
| --- | --- | --- |
| **V_max_** | 0.52 | pmol/min |
| **K_m_** | 585.40 | nM |
| **K_cat_** | 0.432 | /sec |

**Table S3. Comparison of the Cas12a kinetic parameters from different research groups.**

| **Research groups** | **K_cat_ (s^−1^)** | **K_m_ (M)** | **K_cat_/K_m_ (M^−1^ s^−1^)** | **Ref.** |
| --- | --- | --- | --- | --- |
| **Chen *et al.*** | 17 | 1.01 × 10^-6^ | 1.68 × 10^7^ | Erratum to (Chen et al., 2018) |
| **Chen *et al.*** | 1250 | 7.25 × 10^7^ | 1.7 × 10^9^ | (Chen et al., 2018) |
| **Ramachandran *et al.*** | 0.09 | 2.13 × 10^-7^ | 4.22 × 10^5^ | (Ramachandran and Santiago, 2021) |
| **Lv *et al.*** | 0.43 | 5.85 × 10^-7^ | 7.35 ×10^5^ | This work |

**Supplementary Protocol**

**1. Background**

CRISPR Cas12a is an RNA-guided DNA endonuclease enzyme that specifically cuts target DNA. Besides, Cas12a also harbors ssDNA *trans*-cleavage activities, which have been employed for the development of CRISPR-Dx methods such as HOLMES. The definition of Cas12a *trans*-cleavage units uses the fluorometric method, which is based on the generation of fluorescence signals by cleavage of FQ-labelled ssDNA reporters such as the 8C FQ-reporter with the Cas12a *trans*-cleavage activities.

**2. Objective**

To standardize a procedure for measurement of Cas12a *trans*-unit (*trans*U).

**3. Definition**

One Cas12a *trans*U is defined as the amount of Cas12a that cleaves 1 pmol 8C FQ-reporter in 1 min at 37 °C in a total reaction volume of 20 μL.

**4. Reaction formula**

8C FQ-reporter^target-activated Cas12a^> digested 8C FQ-reporter

**5. Materials**

**5.1 CONDITIONS:**

T = 37 °C; Real-Time PCR machine (e.g., QuantStudio^TM^ 3) with λex=488 nm and λem= 535 nm for FAM-labelled reporter.

**5.2 METHOD:**

Continuous Fluorometric Rate Determination.

**5.3 REAGENTS:**

**5.3.1 Cas12a *trans*-cleavage buffer:**

The 10× HOLMES Buffer 1 contained 20 mM Spermidine, 400 mM Tris-HCl, 60 mM MgCl2, 10 mM DTT, 400 mM Glycine, 0.01% Triton® X-100, 4% PEG-20000, pH 8.5 at 25°C.

**5.3.2 crRNA:**

crRNA chemically synthesized and dissolved with nuclease-free water to 1 μM: 5'-AAUUUCUACUCUUGUAGAUUUAUCGCAACUUUCUACUGAAUU-3'.

Note: crRNA can also be synthesized through in vitro transcription with T7 polymerase and purified with the RNA purification kit. The DNA template used should be removed to avoid the generation of background signals in the absence of target DNA.

**5.3.3 LbCas12a:**

LbCas12a Enzyme (10 μM).

(Ordered from Tolo Biotech., Catalog No. # 32108, <https://www.tolobio.com>)

**5.3.4 Target:**

Chemically synthesized oligos, annealed at a ratio of F:R=1:5 in 1× PCR buffer, and finally diluted to 100 nM with nuclease-free water.

T1-50nt-R: 5'-ccgaattcagtagaaagttgcgataacaaaactggccgtcgttttacaac-3'

T1-50nt-F: 5'-gttgtaaaacgacggccagttttgttatcgcaactttctactgaattcgg-3'

**5.3.5 FQ-reporter:**5'6-FAM-CCCCCCCC-3'-BHQ1 (10 μM).

Chemically synthesized and purified with HPLC.

**5.3.6 Nuclease-free water**

**6. Procedure**

**6.1 ESTABLISHMENT AND CALIBRATION OF THE STANDARD CURVE (10 min)**

To establish a standard curve between the amounts of cleaved FQ-reporters and the fluorescence signals, all components but the FQ-reporter are premixed in 1× HOLMES buffer 1, following the formula in the table below. Reporter should be serially diluted, ranging from (0 ~ 1,000 nM), and then added to the reaction system to initiate the *trans*-cleavage reaction. Target-free reaction system should be prepared and labelled as the un-cleaved group, the fluorescence of which is the background signal.

|  | **Cleaved** | **Un-cleaved** | |
| --- | --- | --- | --- |
| **Buffer** (Reagent 5.3.1) | 2.00 | | 2.00 |
| **crRNA** (Reagent 5.3.2) | 0.80 | | 0.80 |
| **Cas12a** (Reagent 5.3.3, diluted to 1 μM by HOMES Buffer 1) | 0.40 | | 0.40 |
| **Target** (Reagent 5.3.4) | 8.00 | | 0.00 |
| **Reporter**  (Reagent 5.3.5, serially diluted) | 8.00 | | 8.00 |
| **Nuclease-free water** (Reagent 5.3.6) | 0.80 | | 8.80 |

After initiation of the *trans*-cleavage reactions, the fluorescence signals are recorded by a qPCR machine till the maximum value is reached in the platform period, and usually 10 min is needed.

The *F_cl_* signal representing the background-subtracted cleaved reporter was generated by subtracting **the signal of a probe-free group** from **the max signal of a corresponding group with titrated probes**. Similarly, the *F_ucl_* signal representing the background-subtracted un-cleaved reporter was generated by subtracting **the signal of a probe-free group** from **the max signal of a corresponding group with titrated probes**. Usually, the probe-free group illuminates extremely weak fluorescence signals and can be directly treated as zero without experimental measurement.

Then, a standard curve of cleaved reporter is drawn with the use of *F_cl_* *versus* *c_cl_*, representing the concentrations (nM) of the cleaved reporter, and the slope *S_cl_* is calculated by a linear fit. Similarly, the standard curve of un-cleaved reporter is drawn with *F_ucl_* *versus* *c_ucl_*, the un-cleaved reporter concentration (nM), and the slope *S_ucl_* is calculated by a linear fit.

The cleaved reporter concentration *c_cl_* (in nM) *versus* time is calculated using Equation (1).


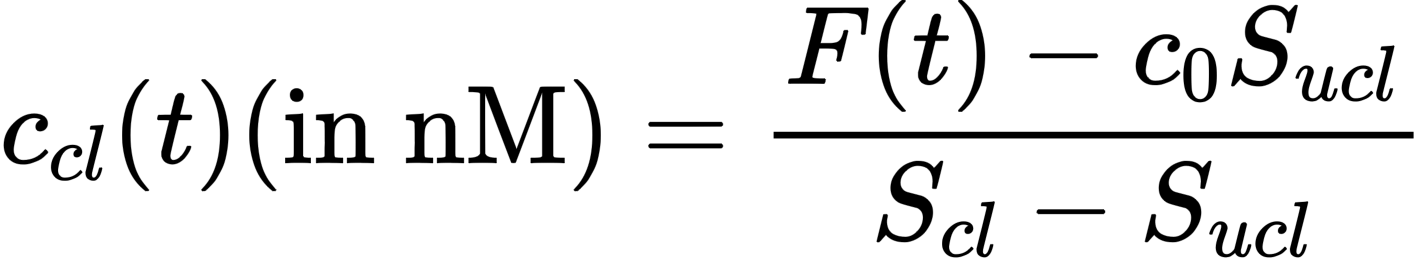
 (1)

*F*(t) is the fluorescence value measured at a certain time, *c_cl_*(t) is the concentration of the cleaved probe cut at that time and *c_0_* is the initial concentration of un-cleaved reporters.

**6.2 CAS12A ENZYMATIC UNIT ASSAY METHOD (30 min)**

To measure the Cas12a *trans*U, 1000 nM ssDNA FQ-reporter is used for analysis, employing Cas12a that is two-fold serially diluted in 1× HOLMES Buffer 1.

First prepare the reagents following the formula in the table below.

| **Reagents** | **volume** |
| --- | --- |
| **Buffer** (Reagent 5.3.1) | 2.00 |
| **crRNA** (Reagent 5.3.2) | 0.04 |
| **Cas12a** (Reagent 5.3.3, serially diluted) | 8.00 |
| **Target** (Reagent 5.3.4) | 0.40 |
| **reporter** (Reagent 5.3.5) | 2.00 |
| **Nuclease-free water** (Reagent 5.3.6) | 7.56 |

Mix the reagents by gentle vertexing for 5 s, followed by brief centrifugation before being aliquoted into optical 8-tube strips (0.2 mL), which are then quickly placed on the qPCR machine to start the Cas12a *trans*-cleavage reaction. The fluorescence signals are recorded for approximately 30 minutes, and the data are then converted into cleaved probe concentrations (nM) with Equation (1). The v_0_ of each reaction is calculated with the online tool (<https://icekat.herokuapp.com/icekat>). The Cas12a specific *trans*-cleavage activities can then be calculated with the following Equation (2).


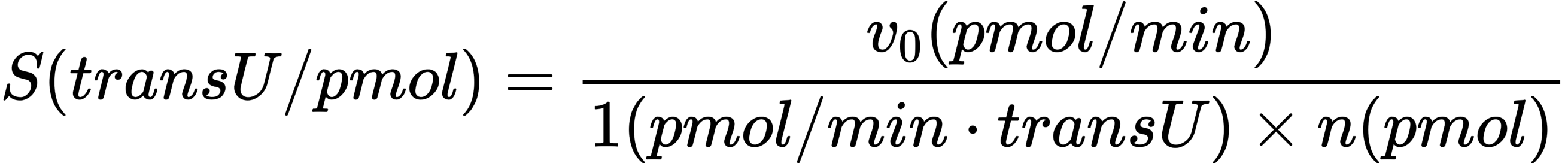
 (2)

Alternatively, if the Cas12a to be tested is not precisely quantitated, its activities can be determined using Equation (3).


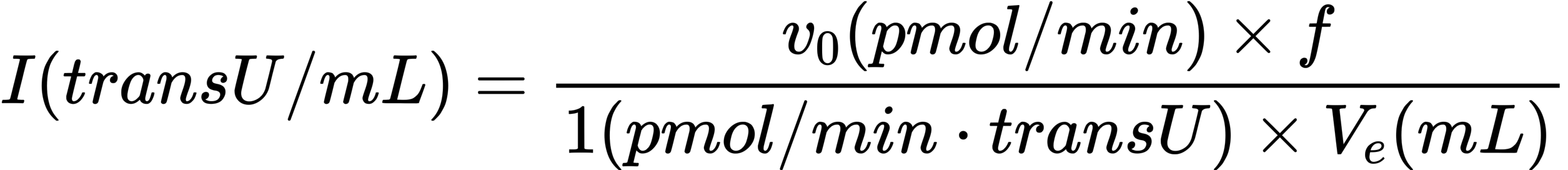
 (3)

In equation (3), f is the dilution factor for Cas12a and V_e_ is the volume of the Cas12a used.

# References

Chen, J.S., Ma, E., Harrington, L.B., Da Costa, M., Tian, X., Palefsky, J.M., and Doudna, J.A. (2018). CRISPR-Cas12a target binding unleashes indiscriminate single-stranded DNase activity. *Science* 360**,** 436-439.

Ramachandran, A., and Santiago, J.G. (2021). CRISPR Enzyme Kinetics for Molecular Diagnostics. *Anal Chem* 93**,** 7456-7464.
